# Participatory Methods to Identify Perceived Healthy and Sustainable Traditional Culinary Preparations across Three Generations of Adults: Results from Chile’s Metropolitan Region and Region of La Araucanía

**DOI:** 10.3390/nu12020489

**Published:** 2020-02-14

**Authors:** Rebecca Kanter, Mariana León Villagra

**Affiliations:** Department of Nutrition, Faculty of Medicine, University of Chile, Independencia 1027, Santiago 8380453, Chile; marianaleonv@gmail.com

**Keywords:** traditional diets, sustainable diets, food variety, Chile, cultural domain analysis

## Abstract

Traditional diets reflect different cultures and geographical locations, and may provide healthy diet options. In Chile, it is unknown whether traditional culinary preparations are still remembered, let alone consumed. Therefore, we adapted methods to identify traditional culinary preparations for healthy and sustainable dietary interventions. In Chile’s Metropolitan Region and the Region of La Araucanía, we collected data on the variety of traditional diets through cultural domain analyses: direct participant observation (*n* = 5); free listing in community workshops (*n* = 10); and pile sort activities within semi-structured individual interviews (*n* = 40). Each method was stratified by age (25–45 year, 46–65 year and ≥ 65 year) and ethnic group (first nations or not). About 600 preparations and single-ingredient foods were identified that differed both in frequency and variety by region. The foods most consumed and liked (*n* = 24–27) were ranked in terms of sustainability for public nutrition purposes. Methods originally designed to collect information about plants of indigenous peoples can be extended to collect data on the variety of existing traditional culinary preparations, globally. Context, both geographical and cultural, matters for understanding food variety, and its subsequent use in the design of healthy and sustainable diet interventions.

## 1. Introduction

Within the last century, the human food system has dramatically changed due to agricultural production and the industrialization of food production. These changes have led to reductions in undernourished people globally, especially in Latin America and Chile [1,2]. Since the 1990 s, however, a nutrition transition has occurred in many countries in which nutrient-poor food consumption has contributed to the global rise in chronic non-communicable diseases (NCDs), such as obesity, type 2 diabetes, and cancers, which account for 70%–80% of worldwide deaths [3].

Given the increasing prevalence of obesity, especially amongst children, in 2012, the Chilean government passed the novel Chilean Law of Food Labeling and Advertising (Law 20.606) that took effect in June 2016, and has since motivated similar regulations worldwide [4]. Law 20.606 advises what not to eat through front-of-package (FOP) warning labels, signaling “high-in” calories, and total sugars, saturated fats, or sodium; and restricts their subsequent marketing and publicity to children (< 14 year). Since Law 20.606 was fully implemented in June 2019, over 80% of all packaged food and beverage products in Chile are predicted to carry at least one FOP warning label [5]. There is a dire need to identify more healthy food and beverage options in Chile and globally. The nutritional and functional value of fresh or minimally processed produce from agricultural production makes said produce a source for healthy foods and beverages [6].

In Chile, the agricultural industry is the second most important economic export sector, but 61% of agricultural employment consists of family farming (AFC, for its acronym in Spanish) that utilizes nearly half of Chile’s agricultural lands, with high involvement in the country’s total production of vegetables (54%), fruits (23%), honey (76%), cattle (54%), and sheep (42%) [7]. Thus, many of the agricultural products consumed by Chileans come from small-scale agricultural producers through sales in *ferias libres* (i.e., traditional outdoor markets), the primary system for commercializing and distributing fresh fruits and vegetables in Chile. Yet, while there are *ferias libres* throughout Chile, on average, only 15% of the Chilean population consumes the World Health Organization’s recommendation of five portions of fruits and vegetables (400 g) per capita per day [8]. Legumes, once paramount to the traditional Chilean diet, used to be widely produced in Chile, but the low legume production reflects the very low average per capita consumption (one to two kilos per capita per year).

Multi-level community-based interventions designed to promote healthy food access and consumption through individual, family, and institutional nutrition education that together ideally lead to behavioral change are recommended in Latin American countries undergoing a nutrition transition [9]. Many countries have also tried to affect individual diet-related behavior changes through food-based dietary guidelines (FBDGs) [10]. The Brazilian and Uruguayan FBDGs are unique, as they promote healthy diets through traditional and homemade food consumption [11,12]. Traditional food systems are defined as, “All food from a particular culture available from local resources and culturally accepted. It includes sociocultural meanings, acquisition/processing techniques, use, composition, and nutritional consequences for people using the food [13].” Traditional diets from different countries have been found to be associated with positive health benefits [14,15,16]. Previous research in Chile suggests that traditional culinary preparations from the *Mapuche* first-nations culture address the environmental, economic, and nutritional dimensions of a sustainable diet in that it originates from times of meat shortages and access to many native vegetable species [17]. Many *Mapuches* in the Araucanía region still acquire fruits and vegetables from *ferias libres* or self-production [18]. Yet, the presence of healthy and sustainable traditional culinary preparations in the diets of Chileans, pertaining to first nations ethnic backgrounds as well as those of different groups is still unknown.

The Chilean FBDGs do not mention the healthy foods that characterize the traditional Chilean diet, but emphasize unhealthy foods not to eat [19,20]. Diverse agricultural production in Chile offers a solution for recovering healthy traditional diets based on fruits, vegetables, and legumes. However, as climate change continues, the sustainability of agricultural production in Chile is increasingly threatened [21]. This confluence of food and nutrition crises with increasing environmental degradation suggests an urgent need for novel analyses and new paradigms to characterize sustainable diets in Chile. Since 2009, research has elucidated both the positive benefits of sustainable diets on human health [22,23,24] and the associations between sustainable diets and climate change mitigation [25,26,27].

In 2019, traditional or “heritage” diets received renewed attention in the scientific community. The EAT-Lancet Commission promoted traditional diets as a potential source of a healthy and sustainable diet [28]. The Journal of the American Medical Association (JAMA) promoted heritage diets as a means for chronic disease prevention [29]. The groundbreaking 2019 report by the Lancet Commission on “The Global Syndemic of Obesity, Undernutrition, and Climate Change” also emphasizes the importance of indigenous and traditional approaches on which to focus greater research regarding public health nutrition related actions related to The Global Syndemic [30]. To date, however, there exists a paucity of studies regarding how to leverage traditional diets for public health, nutrition-related interventions, especially in relation to climate change. A few direct interventions to assess sustainable diets in context have occurred, but are often limited to school settings [31,32,33]. Cultural domain analysis (CDA), a field of cognitive anthropology, has existed for decades. Free lists and pile sorts, two frequently used techniques in CDA, are ways to generate a large amount data from an emic perspective; however, the use of CDA in food and nutrition studies is more common in the food industry and ethnobotany research [34]. Therefore, we aimed to address these research gaps by adapting methods that incorporate CDA to conduct novel research focused on the understanding of traditional culinary preparations that still exist in Chile. Ultimately, we wanted to determine if a variety of traditional culinary preparations exists that can be used to design public health nutrition interventions to promote healthy and sustainable diets, similarly to the FBDGs of Brazil and Uruguay. The incorporation of two main regions in Chile, the central Metropolitan Region (RM) where the capital city of Santiago is located and over half the country’s population resides; and 679 km south of the RM, the Region of La Araucanía (AR) where the country’s largest concentration of first nations people lives, allowing for a greater understanding as to whether food variety data for public health nutrition purposes can be generalized.

## 2. Materials and Methods

### 2.1. Definitions and Overall Approach

The document “Guidelines for procedures for documenting traditional food systems of indigenous peoples: international case studies [13],” referred to as the “toolkit,” was the basis for developing methods to assess healthy and sustainable traditional diets. Aimed at those interested in food-based strategies for nutrition promotion, the toolkit consists of both written procedures and blank forms, to be used in a sequence to facilitate the study of traditional food systems (Table 1). The blank forms are different paper documents that are designed to facilitate the data collection about traditional foods, including food type and related preferences (e.g., taste, consumption). Ideally, the forms should be completed by an anthropologist/ethnographer with experience in food culture studies, with the help of a nutritionist, that together form part of the interdisciplinary research team. These guidelines have been used globally, including indigenous communities in Colombia and Peru [35]. The following definition of traditional food systems included in the toolkit was used: “All food from a particular culture available from local resources and culturally accepted. It includes sociocultural meanings, acquisition/processing techniques, use, composition, and nutritional consequences for people using the food [13].” Meanwhile, the Food and Agriculture Organization (FAO) and Bioversity International define sustainable diets as, “Those diets with low environmental impacts which contribute to food and nutrition security and to healthy life for present and future generations. Sustainable diets are protective and respectful of biodiversity and ecosystems, culturally acceptable, accessible, economically fair and affordable; nutritionally adequate, safe and healthy; while optimizing natural and human resources [36].” Thus, the definition of sustainable diets consists of five criteria: culturally appropriateness, neutral or positive environmental impacts, physical accessibility, economical accessibility, and nutritional adequacy.

For the determination of both traditional and sustainable diets in Chile, we adapted the methodologies defined by Kuhnlein et al. by: (1) extending the assessment of individual plant species to individual foods, or culinary ingredients and culinary preparations, consisting of multiple culinary ingredients; and (2) considering the aforementioned sustainable diet criteria throughout the methodological development and its subsequent implementation. The newly adopted methods, described below, were designed to collect information from adults (≥ 25 year), considering age group (25–45 year, 46–64 year, ≥65 year) and ethnicity (i.e., from first nations or others). Therefore, prior to data collection, the blank forms in the toolkit used in this study were slightly modified, including translation into Spanish and eliminating sections directed at children (Table 2). The data collection in the Metropolitan Region took place between December 2017 and August 2018, whereas the data collection in the Region of La Araucanía took place between December 2018 and September 2019. All participants provided written informed consent for inclusion prior to their participation in the study. The study was conducted in accordance with the Declaration of Helsinki, the International Ethical Guidelines for Health-related Research Involving Humans CIOMS 2016, and the ICH Guidelines for Good Clinical Practice 2016. The data collection was approved by the Ethics Committee for Human Subjects Research at the University of Chile’s Faculty of Medicine, application number 164−2017.

### 2.2. Overall Study Site Selection

The study sites in which the research activities were conducted were at the county level. Prior to conducting the participatory research activities, we used purposive sampling to select counties that were both geographically and socio-demographically diverse. Specifically, we considered area type (urban/rural; central, periphery), ethnicity (first nations prevalence or not), and socio-economic level in site selection. The socio-economic level was based on an ethnographic perspective rather than fixed socio-economic levels used for governmental purposes. There was not an a priori target number of study sites or counties in the research design. To help determine where to conduct research in the Metropolitan Region (RM), data from the geographic distribution of public family health centers was used as a first step from which to select a diverse range of counties (*comunas*) (Table 3). In contrast, in the Region of the La Araucanía (AR), a greater emphasis was placed on the unique types of geographical terrain from which to select a diverse range of counties (Table 3). In total, research activities were conducted in nearly half of all provinces (*n* = 3/6) and counties (*n* = 25/52) in the RM; and both provinces and 43% of all counties in the AR (*n* = 14/32).

### 2.3. Cooking Observations and Free Listing of Traditional Culinary Preparations

From each region, we purposively selected five key informants to capture cooking methods and traditional culinary preparations across age (25–45 year, 46–64 year, ≥ 65 year) and ethnic groups (i.e., from first nations or others). Each key informant meeting consisted of two components over about 2.5 h: (1) direct participant observation while cooking a traditional culinary preparation [13]; and (2) a free listing activity of traditional culinary preparations. The research assistant called each key informant to confirm the recipe they were planning to prepare to see if it was unique from the previously prepared recipes, and provided a fixed amount (USD 20.00, or CLP 15.000) for the ingredients.

Key informants ranged from professional chefs (*n* = 3), a nanny (*n* = 1), housewives (*n* = 2), an executive secretary (*n* = 1), an entrepreneur (*n* = 1) to food aficionados (*n* = 2); and only one was male. The key informant meetings took place in home settings to observe the present-day kitchen. We conducted 8 key informant meetings in participants’ homes, one in a key informant’s restaurant kitchen, and one in a participant’s friend’s home due to a problem with their own kitchen. During each key informant meeting, the research assistant directly observed how the key informant prepared the traditional recipe, taking written notes and photographs. A digital recorder was used to record all key informant meetings. Often after or during the cooking process, depending on the recipe chosen, the research assistant conducted a free listing activity by asking the key informant to freely list what they believed to be traditional culinary preparations, including individual food items (e.g., pears). If the key informant asked what was considered “traditional,” the research assistant provided little guidance. The free listing activity provides a baseline set of traditional culinary preparations pertinent to a country that are still remembered without probing. Each key informant was also asked during what time range they consumed each weekday and weekend meal (i.e., breakfast, lunch, and dinner or *once*, a lighter evening meal that many Chileans eat instead of dinner which often consists of bread, avocado, and black tea). After each key informant meeting, the research assistant reviewed and filled in gaps in field notes. All key informants were given USD 20.00 (CLP 15.000) cash as compensation for their time.

After the key informant meetings, all hand recorded data were transcribed and imported into Atlas TI Version 8.1. All free listed traditional culinary preparations were coded according to mealtime (if applicable) and food subcategory (e.g., soups). All traditional culinary preparations identified during the key informant interviews were subsequently used to guide the community workshops in the same region as described in the following subsection.

### 2.4. Community Workshops

To further identify traditional culinary preparations by mealtime and understand if these traditional culinary preparations are still liked and consumed by the public, we conducted ten community workshops, two for each age and ethnic group included in this study. In total, 73 men and women participated in the workshops in the Metropolitan Region, and 67 participated in the Region of La Araucanía. The community workshops were referred to as group interviews in Spanish to avoid confusion with cooking or nutrition education workshops. Each workshop was planned for a minimum of 6 people, but one workshop per region had a minimum of 4 people, and they had a maximum of 10. The ideal number of participants is 8 for optimal management and participation by all, given the time provided.

There were no inclusion criteria other than being in the designated age or ethnic group for a workshop. Adults without expert knowledge in culinary preparations were encouraged to participate. The physical sites for the workshops were socio-economically and geographically diverse (Table 3). For example, we selected adult geriatric day care centers from both upper-middle and lower-middle class areas for the two community workshops of those ≥65 y. Workshop participants were recruited using different means, from word of mouth to having a center recruit their community.

Initially, we aimed to fully replicate the community workshop process outlined in the toolkit (Table 1), but doing so required the research assistant to heavily control the discussion to administer all the forms. Consequently, we perceived that participants felt pressured to remember, or respond, which inherently pushed participants to only mention “everyday” or recently consumed foods, rather than “traditional foods” per se. Therefore, we did not include the data collection forms about foods rarely prepared or consumed. Instead, we included some discussion questions about foods from “the past.” In each workshop, there was only enough time to collect additional data about some of the preparations discussed (Table 2: form 2.4). After the research assistant obtained informed consent, each workshop had a duration of three hours. The research assistant used a brief protocol as a guide for the use of materials and methods within the time allotted (see Appendix A).

The community workshops also include a free listing activity of traditional culinary preparations. Specifically, two large posters (122 cm × 91 cm) were designed for the free listing activity—one poster for the preparations for breakfast (*n* = 15 rows) and lunch (*n* = 25 rows); and the preparations for dinner (*n* = 20 rows) and *once* (*n* = 20 rows) on the other. Each row was followed by columns denoting frequency (daily, ≥1/month, only holidays, never) and seasonality (in winter, in summer, or both) of consumption. Each community workshop began with introductions and a clarification that we were not seeking detailed recipes. The research assistant then asked the group to freely list, by mealtime, what they considered to be traditional culinary preparations and recorded them verbatim onto the posters facing the group.

After the free listing activity, the group was asked who still consumes each preparation listed, independent of frequency of consumption and likeability (taste); and the number was recorded in parentheses next to each recorded preparation. For each traditional culinary preparation listed, the likeability, frequency, and seasonality of consumption (winter/summer) was also determined. Participants were each given a different colored permanent marker and asked to divide into two groups, each group forming a single-file line in front of a poster. Guided by the research assistant at one poster and another project member at the other poster, the participant at the front of the line was asked to mark the columns that correspond to the frequency (daily, daily, ≥1/month, only holidays, never) and seasonality (in winter, in summer or both) of consumption for the first culinary preparation recorded on the poster; and then to return to the back of the line. This process repeated until all participants filled out the frequency and seasonality of consumption for each preparation written on both poster forms. Taken together, each poster was a hybrid of forms 2.2 and 2.3 from the “toolkit” by Kunhlein et al. (Table 1; Table 2: forms 2.2–2.3).

Following the dynamic process to collect the food frequency and seasonality data, the research assistant then guided a discussion to collect more information regarding specific preparations (from *n* = 1 to *n* = 22, depending on time) listed on the poster forms (Table 2: form 2.4). Often, the additional data were collected for preparations unique to that workshop (i.e., not mentioned in previous workshops); unfamiliar (i.e., not an obvious preparation like scrambled eggs); or familiar, but whose preparation varies by socio-demographic context (e.g., how soup varies globally).

Each workshop ended with a group discussion on the likeability, or taste preference, of each preparation listed, again independent of consumption frequency. The research assistant recorded the number of people who really liked a preparation with a green marker on the poster, and then, using a red marker, recorded how many strongly disliked the same preparation. After each community workshop, the research assistant reviewed and revised the field notes. All participants were compensated with approximately USD 7.00 (CLP 5.000) cash.

### 2.5. Generation of the “Short List” of Traditional Culinary Preparations for Targeted Intervention Purposes

The free listing activities in the community workshops generated an exhaustive list of hundreds (> 800 records per region) of traditional culinary preparations that are both unique and often repeated within or between regions. Therefore, we adapted a recommendation by Kunhlein et al. (Table 2: form 2.6) to identify a “short list” of 25–30 preparations with the greatest potential for inclusion in future public health nutrition initiatives. To do so, the project team performed, and revised, double data entry of the workshop poster forms into a customized online data platform [37]. To identify the variety of unique foods and beverages listed throughout the workshops, data management was performed to aggregate similar, repeated preparations into one record (e.g., tomato and tomatoes); and then aggregate the unique preparations into subcategories (e.g., coffee/tea/maté or eggs/with). After the aggregated database of traditional culinary preparations (RM: *n* = 651; AR: *n* = 592) was organized into subcategories (RM: *n* = 106; AR: *n* = 112), the top 70 preparations still usually consumed on a daily basis and those highly liked were extracted to create the short list. From the top 70 preparations still usually consumed or highly liked. The subcategories of culinary preparations that were deemed to be “not traditional” (*n* = 4), such as oatmeal or yogurt; “not healthy” (*n* = 18), such as fried desserts; or “not sustainable” (*n* = 2), such as heavy meat dishes, were excluded. Because many of the top still usually consumed preparations were also the highly liked preparations, after these exclusions, the short list was derived from the top 24 (in the RM) and the top 27 (in the AR) most usually consumed and highly liked dishes.

### 2.6. Sustainable Diet Estimation of Traditional Culinary Preparations: Pile Sorting Activities

To carry out the pile sorting activities, customized laminated index cards (16 cm by 12 cm) were designed, each with a photo of a traditional culinary preparation from the short list. For some preparations it was hard to find freely available quality images. Thus, the research assistant cooked, and subsequently, photographed the culinary preparation. In both regions, five pilot interviews were conducted (one per age and ethnic group) to ensure that participants could quickly identify, with minimal help, all the photos for the pile sorting activities. The overall objective of the pilot interviews was to determine what images to modify or add, such as what is meant by “fruit,” “salads,” or “legumes,” whose meanings initially, were conveyed through written text, rather than a photo. For example, for fruit, based on participant preference to consume either whole fruits or fruit salad, the card was divided down the middle. A few small images of individual fruits (apple, banana, orange, pear) were placed on one side, and a picture of a fruit salad with Chilean berries was placed on the other. Following the pilot interviews, about half of the photos were replaced with different, more representative images.

To determine the perceived sustainability of the preparations on the short list, we conducted 40 semi-structured individual interviews; specifically, 8 interviews per age (25–45 year, 46–64 year, ≥65 year) and ethnic group (i.e., from first nations or others). Each individual 90 min, digitally recorded interview included three separate components based on the previously mentioned definition of sustainable diets by the FAO and Bioversity International [36], as well as the toolkit [13]: (1) 5 pile sorting activities (60 min); (2) a shortened food-frequency questionnaire (15 min); and (3) a taste likeability questionnaire (15 min) (Table 2). During each pile sort activity, participants were asked to group cards (*n* = 2 to 10 times) based on the positive and negative aspects for each of the five sustainable diet criteria. Participants could ask the research assistant to add any card they felt necessary to any of the five pile sorting activities. For example, many participants had difficulty grouping the cards without a “bread” card, and so the assistant wrote the word “Pan” (bread, in Spanish) on a blank card for participant use. After each pile sort activity, the research assistant asked about the cards that the participants chose to not include in sorted piles.

Participants first sorted the cards based on “menus” that they themselves would or might eat; “menus” that family members (e.g., partner, children) might eat; and what they thought “another” person (like a neighbor, someone representative of their community or environment) might eat, independent from what they or others in their household currently eat. Next, participants pile sorted the cards according to positive and negative environmental impacts; and the research assistant was instructed to not give examples or hints about what was meant by the environmental impacts of food. If asked, the research assistant provided reassurance that there was no right or wrong answer, and restated the question by using a different word to environment: ecosystems in the Metropolitan Region; and nature in the Region of La Araucanía. Participants were then asked to make pile sorts in terms of physical accessibility (i.e., within the physical environment of their normal weekly routine), regarding either the culinary preparation itself (e.g., is it offered at a restaurant?), or its individual ingredients. The research assistant directed participants to focus only on physical accessibility, which was often confused or combined with economic accessibility, by defining physical activity as being close to where one travels weekly, and independent from preparation cost, signaling that this was the next pile sort activity. Thus, in the fourth pile sort activity, participants were asked about the economic accessibility of each culinary preparation, both in terms of the culinary preparation as a whole (e.g., can they afford to purchase the dish at a restaurant) and its individual ingredients for home preparation. The final pile sort activity was about each participant’s perception of the nutritious qualities, or lack thereof, of each culinary preparation. Participants were asked how each person included in the first card sort activity (e.g., themselves, family members, and others) might perceive certain culinary preparations as nutritious and others as not nutritious.

The food frequency and taste questionnaires asked about all traditional culinary preparations on the short list, even if a participant had not included a preparation(s) in the pile sorting activities. The short food frequency questionnaire (Table 2: form 4.8) asked about the frequency of consumption (daily, > once a month, once a week, special occasions, or never) and the usual portion size as an open-ended question. The taste acceptability questionnaire (Table 2: form 4.2) gauged the participant’s taste preference for each culinary preparation based on face icons representing a Likert scale (i.e., 5 = really like a lot, 4 = like, 3 = indifferent, 2 = do not like, 1 = really do not like). After completing the taste acceptability questionnaire, participants were asked if they had anything else to add before ending the interview. After each interview, the research assistant revised and annotated the field notes for completeness. Except for two instances in which the interviews were conducted in public (café) settings, all interviews were conducted in people’s homes, taking advantage of a private setting and a large table space on which to pile sort. Professional transcribers transcribed interviews verbatim. All participants were compensated with approximately USD 7.00 (CLP 5.000) cash.

### 2.7. Sustainable Diet Estimation of Traditional Culinary Preparations: Sustainable Diet Score

To quantitatively estimate the perceived sustainability of the 24–27 traditional culinary preparations based on the data from the pile sorting activities, we created a sustainable diet score. One point was awarded for each positive characteristic related to sustainability; and the total tabulated per criteria per person. For each preparation, a sustainability score was calculated as the proportion of sustainability dimensions (x) for which it had positive criteria ((x/5) *100%). For example, a preparation would have perfect (100%) sustainability for a given participant if that participant had identified positive characteristics for each of the 5 sustainable diet criteria for that preparation. Conversely, a culinary preparation would have zero (0%) perceived sustainability if a participant did not identify any positive characteristics for that preparation. The most common example being when a participant perceives positive characteristics for all of the 5 sustainable diet criteria, except environmental impact: that yields an 80% (*n* = 4/5) sustainability score. The average sustainability score was calculated for each of the 24–27 culinary preparations by averaging, over the 40 participants, the individual sustainability scores, and ranking the average scores in descending order from 100%.

## 3. Results

### 3.1. Main Findings from the Key Informants

All key informants took pride in the recipes they prepared. Based around a variety of local ingredients, reflecting the local socio-cultural realities, the preparations varied within and between regions (Supplementary Figure S1). The Chilean stew Charquicán was prepared three different ways, once in the Metropolitan Region (RM) and twice in the Region of La Araucanía (AR). In total, the free listing exercise yielded 220 unique traditional culinary preparations in the RM and 351 in the AR. The most common overarching food categories of the free listed preparations were: breads (RM: 8%, AR: 7%); vegetables/dark green leafy vegetables (RM: 6%, AR: 10%); sandwiches (6%); sweets and marmalades (AR: 6%); soups and stews with meat (RM: 6%) and without meat (AR: 6%); beans (RM: 4%); and potatoes (AR: 4%). While all key informants could identify many traditional dishes, it was clear that all key informants did not consume the traditional dishes they had free listed on a regular basis. All key informants asked about the meaning of “culinary preparations/traditional dishes,” and seldom associated traditional culinary preparations to specific mealtimes (e.g., lunch).

### 3.2. Main Findings from the Community Workshops

In both regions, hundreds of unique traditional culinary preparations were identified during the community workshops (about *n* = 600 per region) which pertained to food and beverage subcategories both shared and unique between regions (Table 4). In contrast to the key informant interviews, the community workshop participants were inherently pushed to free list the traditional culinary preparations by mealtime. However, it was difficult for the community workshop participants to think in terms of a “menu” or eating times (breakfast, lunch, *once*, dinner). From this free listing activity, it is clear dinner is not a common mealtime. Participants were capable of making distinctions between the traditional dishes of “today” versus “the past.” In the conversations about specific traditional culinary preparations (Table 2: form 2.4) a richer dialogue and space of reflection occurred than during the free listing activity. As a result, additional themes arose that included: (1) temporal changes in the daily work schedule due to changes in the labor system (e.g., a shift from agricultural to service labor); (2) seasonality; (3) perceptions about gender; (4) stigma related to preparations being “poor people’s food” yet longed for at the same time; and (5) the changes in the costs of certain ingredients between “now” and “then,” among others. Finally, in the RM, the conversation around “taste preference” worked well to end the workshop, while in the AR it was included in the middle of the workshop to maintain the workshop momentum.

### 3.3. Main Findings from the Pile Sorting Activities

The traditional culinary preparations identified as the most sustainable in the RM were: fruits (91%), salads (90%), scrambled eggs with tomato/onion (82%), vegetable soup (78%), and legumes (78%) (Table 5). Fish soup (52%), shredded beef (48%), and empanadas (39%) were identified as the least sustainable. In the AR, the traditional culinary preparations identified as the most sustainable were salads (94%), scrambled eggs (92%), and homemade bread (90%); and soup with mussels or mixed seafood (63%), salad with *diguenes* (61%), and large pine nuts (54%) were identified as the least sustainable. Of the five sustainable diet criteria, the environment dimension was the most difficult for participants to think about in relation to diet, and thus, verbally expound upon. Two-thirds of participants thought that no dish produced any environmental impact. In the RM, those that did associate negative environmental impacts with dietary intake, erroneously confused environmental impact with health problems or with household contamination from cooking fried foods. Those in the AR, accustomed to composting, tended to positively associate all the culinary preparations with the environment. The livestock and fishing industries were often cited in both regions as having negative environmental impacts. A recurring theme throughout the pile sort activities was that that the participants felt that “they were missing something” regarding the cards used in the pile sort activities. The most common extra cards that were incorporated during the pile sort were the lack of bread and sides (e.g., rice). However, participants almost never commented about the lack of beverages included in pile sort cards. As a result of the pile sort activities, it was possible to rank the average perceived sustainability of each of the traditional culinary preparations on the short list, which in turn can be used for future interventions and studies.

## 4. Discussion

This study enriched previously published methods by Kunhlein et al. [13] to study healthy and sustainable traditional culinary preparations in Chile. Direct observation of adults of different ages and ethnic groups cooking different traditional culinary preparations generated a warm and trustworthy dynamic between the interviewer and the participant. The key informant interviews also led to a rich collection of photographs regarding traditional cooking methods and ingredients that may eventually become lost. Free listing in both individual and workshop settings yields a large number of traditional culinary preparations that may not necessarily be still consumed on a regular basis but are still remembered. The traditional culinary preparations generated from the free listing activities varied both between and within regions, suggesting the importance of local context in understanding the healthy traditional culinary preparations that are still commonly consumed and liked. Finally, the pile sort activities helped elucidate how Chilean adults in two different regions conceptualize, and thus, perceive, the five dimensions of sustainable diets in relation to traditional Chilean culinary preparations. Taken together, it is apparent that the recalling of traditional Chilean culinary preparations is possible and varies by region.

The sheer quantity of data produced by free listing, while it could be perceived as overwhelming, is necessary for understanding the food variety in a given area. The food variety mentioned during free listing is largely independent from the packaged food and beverage supply pervasive in the supermarket setting, at least in Chile [5]. The challenge remains of how to group individual preparations or foods and beverages listed into both meaningful and useful groupings. The fact that the subjective subcategories used in our study to group the large variety of traditional culinary preparations listed were not all the same between regions, elucidates the importance of implementing similar research methods to assess food variety in different geographical locations, rather than assuming that food variety is consistent between regions of a country. A common critique of cultural domain analysis (CDA) is that it elicits data of the most familiar items. However, it is clear from our study results that CDA also generates a large amount of data in which one can discriminate between the food variety in two distinct geographical regions. Thus, it is evident that context matters in both understanding and utilizing food variety for eventual public health nutrition purposes.

The primary strength of this study is that we expanded upon pre-existing participatory methods to collect data on traditional and sustainable culinary preparations that can be used for public health nutrition purposes. The qualitative research methods employed provided information with fewer biased assumptions imposed by the investigator than quantitative research. The open-ended and flexible nature of free listing and group discussions, taken together, can be more revealing when the topic under study is complex and cannot be clearly quantified, such as traditional culinary preparations [38,39,40]. Another study strength is that the participants expressed that they liked taking part in the research activities, which suggests veracity in the data collected. The strength of having a short list of traditional culinary preparations ranked by perceived sustainability is that it can inform both sustainable food based dietary guidelines and other public health nutrition initiatives related to sustainable diets.

The main limitation of a short list of traditional culinary preparations ranked by perceived sustainability is that the five dimensions of sustainable diets are perceived, rather than quantitatively assessed. For example, it is possible that when similar methods are applied to other geographical locations that the traditional culinary preparations identified as most sustainable are not most sustainable according to other measurement criteria for sustainability, such as greenhouse gas (GHG) emissions; or that people perceive certain preparations to be “healthy” that according to other nutritional criteria are identified as “unhealthy.” Results from two different regions in Chile suggest that adults are capable of discriminating between more and less sustainable culinary preparations. The achieved results can be compared to other indicators of sustainable diets. However, the local reality must be taken into account. For example, if an animal-based culinary preparation is defined as more sustainable by a different indicator of sustainable diets (e.g., the GHG emissions of lamb), but that type of animal is not financially or physically accessible to most in the local area, what is the utility of promoting this culinary preparation through a public health nutrition initiative?

In carrying out the aforementioned methods in two distinct local regional contexts, there were both methodological and practical challenges. The strength of using an existing toolkit was also a limitation in that we applied methods designed for studying small groups of peoples to broader public health purposes, and also extended the focus on the identification of plant species to culinary preparations. The adoption of the methods to culinary preparations and the common mealtimes in Chile (breakfast, lunch, dinner, *once*) did not consider a priori other “mealtimes” such as “snacking” (“*picoteo*”) or the hybrid “*once*-dinner.” Furthermore, while the mixed methods approach outlined in the toolkit led to a unique participatory experience that may be considered a study strength, it may also be a study weakness. During the key informant interviews, sometimes, it was challenging to both observe and take notes about the recipes being prepared. In particular, when some key informants had a tendency to converse with the observer at the same time about topics not related the recipe being prepared or cooking (e.g., personal issues). During community workshops, there was not enough time to generate a dynamic of more fluid conversation, or collect all the data suggested in the toolkit. The balance between qualitative and quantitative methods was always a challenge, given each has biases.

Furthermore, we encountered practical challenges unique to the community workshops. First, we quickly realized that it is nearly impossible to conduct activities during Chilean summertime (i.e., the entire month of February), when many are on vacation and public buildings are often closed. Second, there were challenges to recruiting community workshop participants. Specifically, the more “well-off” seemed to not want to utilize free time for study activities that were not of maximum personal interest. The 46–65 year age group was the hardest to recruit and to engage during the workshops. Third, community workshop participants often showed up late. Therefore, we suggest that others implementing similar study methods should budget for and include sufficient time for unanticipated tardiness. A fourth challenge unique to the community workshops was not having enough human resources: ideally, a research assistant plus two additional assistants to help with note taking and workshop facilitation. The fifth and final challenge during the community workshops was related to contextual challenges about the socio-demographic makeup of the group. In both regions, when working with older adults (> 65 years), it was important to be more patient, conduct each workshop at a slower pace, and make an extra effort to inquire about traditional culinary preparations of the “past” (i.e., from when they were children). In the Region of La Araucanía, those working in more rural or peri-urban settings had a different rhythm and conceptualization of time than those in the urban Metropolitan Region. As such, we had to change the timing and organization of the community workshops, but not the data collection tools.

The approaches taken by other researchers in the study of food variety for the design of public health nutrition interventions differ from our approach for two main reasons. First, food variety is frequently measured and assessed as it pertains to the food environment, either pertaining to a specific geographical space (e.g., neighborhood) or type of retail store (e.g., small corner stores), or both [41,42]. Second, when food variety is assessed within the scope of traditional diets for potential health purposes, it appears to be often only with regard to the Mediterranean diet [43], a diet that is not sustainable in many places of the world. To the best of our knowledge, however, there are no other previously published methods to assess traditional diets in any context that exists for the primary aim of promoting healthy and sustainable diets. Therefore, the methods suggested by Kunhlein et al. through the toolkit and our novel adaptation of these methods is necessary for the determination of sustainable traditional culinary preparations in local contexts. Taken together, our findings, or those from a similar adaptation, and subsequent implementation of these methods, can and should be used to collect data on food variety that can be used to inform public health nutrition interventions related to sustainable diets.

## 5. Conclusions

Traditional culinary preparations still exist in the memories of Chileans in two distinct regions. The finding that vegetable-based traditional preparations make up the greatest prevalence of recalled dishes is important for understanding the current food environment to design healthy and sustainable diet interventions. The implications of utilizing methods originally designed to collect information about traditional food systems of indigenous peoples can be extended to collect data related to healthy and sustainable traditional culinary preparations for public health nutrition purposes in the Chilean context. In conclusion, multiple components of cultural domain analysis, such as free listing and pile sorting activities, generate a large amount of data on food variety that can be used for the future design of applied public health nutrition intervention approaches aimed at promoting healthy and sustainable diets.

## Figures and Tables

**Table 1:** Key features and brief summary of Guidelines for Procedures for Documenting Traditional Food Systems of Indigenous Peoples: International Case Studies.

Step	Objective	Rationale	Forms
Prepare interdisciplinary research team	Collect background data	To have experts in local leadership; food culture anthropology; food analysis; food and dietary databases	None
Gather data on traditional foods	Create a list of traditional food species	To provide the foundation for the project	2.1, 2.2, 2.3, 2.4, 2.5 ^2^
Select a short list of potential foods for more focused study		To identify 25–30 foods with good potential for nutrition intervention purposes	2.6
Determination of food items for analyses	To obtain scientific parameters of traditional foods with missing data ^1^	Some food items may need taxonomic identification and nutrient analyses	3.1–3.4
Individual interviews	To understand traditional food use and its relation to taste and consumption preferences	To better understand how the short list of traditional foods are used in the community and attributes people attach to them	4.1 A & B, 4.2, 4.8 ^2^
Intervention planning	To plan for a food-based intervention to improve public health nutrition	To review data collected in the previous steps To consider the local advantages and constraints to using the short list of traditional foods for intervention purposes To work with the community to: (i) develop and implement an intervention; and (ii) measure intervention outcomes	5.1

^1^ Not included in this study. ^2^ This objective includes forms not mentioned, targeted at children and micronutrient intake, which were excluded from this study.

**Table 2:** Summary modifications to study instruments compared to the original “toolkit” ^‡^.

Research Activity (Human Resources, *n* = )	Toolkit Form	Modified form Applied (Time Allotted)	Modifications
Key informant interviews with free listing and direct observation: *n* = 1, research assistant (RA)	2.1	Modified 2.1 (2.5 h)	Based on initial key informant interviews, for the purpose of this study form 2.1 was modified slightly and included columns for identifying the mealtime(s) for the listed preparation (e.g., breakfast, lunch and/or dinner) as well as a column for ‘side dishes’ that might accompany the preparation (if applicable). Compared to the original form 2.1, additional space for the ‘local name/national language name’ was not included beyond the name listed in the first column of the form.
Community workshops with free listing: *n* = 3, 1 RA guiding and moderating the discussion; 1 person recording the preparations and associated comments on the related forms and 1 person to control time and record the social dynamics, such as how the space is used	2.2	Hybrid 2.2–2.3 (90 min)	Rather than collect data separately on forms 2.2 and 2.3, respectively, we created one hybrid form: by adding a fourth food group section; and removing the columns for comments and
	2.3		The columns for months, likeability and comments on form 2.3 were removed. And the columns related to consumption frequency and seasonality were added.
	2.4	2.4 (40 min)	We slightly modified form 2.4 from its original version designed for plant species. Specifically, we removed a section on nutrient composition and kept the remaining open-ended questions about notes regarding the species under question (in our case a culinary preparation), how the preparation ingredients are obtained (e.g., bought, harvested, etc.), which ingredients are more likely to be harvested at home or bought, seasonality of consumption, estimated cost of the preparation (if known). We also slightly modified the “use/price” table on the second page of form 2.4 to be two separate tables, one for preparation use by month and one for price by month, respectively.
Select a short list of potential foods for more focused study	2.6	2.6	We slightly modified form 2.6 from its original version designed for plant species; to list 25–30 traditional culinary preparations.
Individual semi-structured interviews with pile sorting: *n* = 1, RA	4.1 A	4.1 A (1 h)	We expanded the number of tables for card sort groupings from two to five–one for each of the five criteria used to define sustainable diets; and added two additional final columns to this form, one column full of plus signs and a column full of minus signs. The plus and minus signs were added to form 4.1 A, so that the research assistant could circle if the card grouping corresponded to a ‘negative’ or ‘positive’ impact according to the participant. For example, when the interviewer asks the participant to group the cards according to negative environmental impacts the interviewer circles the minus sign at the end of this row. The qualification of each card sort, or row, as either positive or negative was important for more quickly tabulating the interview results by sustainable diet criteria without having to listen to audio files or wait for interview transcriptions.
	4.2.	4.2 (15 min)	We slightly modified the form 4.2 by removing the columns for children.
	4.8	4.8 (15 min)	We modified the form 4.8 by removing the information about the child; and removed the columns to assess micro-nutrients. Instead, form 4.8 consisted only of the brief food-frequency questionnaire related to the foods on the short-list (form 2.6).
	4.1 B	4.1 B (7 h, 10 min/form)	The sustainable diet score was based on summarizing the card-sort responses (form 4.1 B) from the form 4.1 A. Therefore, we constructed our version of form 4.1 B in Excel with each row representing one of the culinary preparations (i.e., cards) and each column representing each of the five sustainable diet criteria (i.e., card sort activity themes).

^‡^ See Table 1 for information on the original toolkit. Modified forms (in Spanish) available upon request.

**Table 3:** Study site territorial distribution overview by region.

Territory	Province	County	Key Informant Interviews	Community Workshops	Individual Interviews	Total by Territory
**Metropolitan Region**		***n* = 25**	***n* = 5**	***n* = 10**	***n* = 40**	***n* = 6**
North	Santiago	Conchalí	0	0	2	5
		Huechuraba	0	0	1	
		Recoleta	0	0	2	
Center	Santiago	Cerillos	1	0	0	13
		Maipu	0	0	4	
		Estación Central	0	0	2	
		Santiago	1	1	4	
East	Santiago	Las Condes	0	0	1	17
		Peñalolen	0	0	1	
		La Reina	0	0	1	
		Providencia	1	1	0	
		Ñuñoa	0	2	6	
		Macul	1	1	2	
South	Santiago	San Miguel	0	0	1	3
		La Cisterna	0	0	1	
		La Granja	0	0	1	
West	Santiago	Quinta Normal	1	0	0	12
	Santiago	Lo Prado	0	2	1	
West	Melipilla	Curacaví	0	0	3	
West	Talagante	Padre Hurtado	0	1	1	
	Talagante	Talagante	0	1	0	
	Talagante	Peñaflor	0	0	1	
	Talagante	El Monte	0	0	1	
Southwest	Santiago	La Florida	0	1	2	5
Southwest	Mountain	Puente Alto	0	2	2	
**Region of La Araucanía**		***n* = 14**	***n* = 5**	***n* = 10**	***n* = 40**	***n* = 7**
Valley—Capital city	Cuatín	Temuco	2	2	10	15
		Nueva Imperial	0	1	0	
Valley—urban	Cautín	Padre Las Casas	0	2	5	7
Valley Mirco- Climate—Nahuelbuta	Malleco	Angol	1	1	2	4
Valley	Cautín	Freire	1	0	0	11
	Malleco	Traiguén	0	2	2	
	Cautín	Gorbea	0	0	5	
	Cautín	Labranza	0	0	1	
Coastline	Cautín	Queule	1	0	2	8
	Cautín	Carahue	0	1	1	
	Cautín	Toltén	0	0	2	
Mountain	Cautín	Cunco	0	2	3	5
Mountain—Lake	Cuatín	Lican Ray	0	0	3	6
		Villarrica	0	0	3	

**Table 4:** Frequency of traditional culinary preparations mentioned during the community workshops by region ^†^.

Metropolitan Region		Region of La Araucanía	
Subcategory	*n* = (%)	*n* = (%)	Subcategory
COFFEE/TEA/MATÉ	42 (6.5)	23 (3.9)	EGGS/WITH
EGGS/WITH	28 (4.3)	22 (3.7)	COFFEE/TEA
BREADS	27 (4.1)	22 (3.7)	SOUPS
WITH VEGETABLES	25 (3.8)	21 (3.5)	WITHOUT A SUBCATEGORY
WITH CHEESE	19 (2.9)	16 (2.7)	WITH *COCHAYUYO*
WITH MEAT	18 (2.8)	16 (2.7)	*SOPAIPILLAS ETC*
LEGUMES	18 (2.8)	15 (2.5)	POTATOES
BEVERAGES (ALCOHOLIC)	17 (2.6)	14 (2.4)	FRUIT
*CAZUELAS*	15 (2.3)	13 (2.2)	MILK WITH…
WITH SAUSAGUES	15 (2.3)	12 (2)	*CAZUELAS*
SOUPS	15 (2.3)	12 (2)	LEGUMES
WITH AVOCADO	14 (2.2)	12 (2)	BREADS: NOT SPECIFIC
FRUIT	14 (2.2)	12 (2)	SWEET BREADS/CAKES
YOGURT	14 (2.2)	11 (1.9)	BROTHS
SWEET BREADS/CAKES	13 (2)	11 (1.9)	WITH CHEESE
POTATOES	12 (1.8)	11 (1.9)	*EMPANADAS*
FISH	12 (1.8)	10 (1.7)	BEVERAGES (ALCOHOLIC)
PASTAS	12 (1.8)	10 (1.7)	WITH SAUSAUGES
TUNA OR MACKEREL	11 (1.7)	10 (1.7)	TOMATO
BROTHS	11 (1.7)	9 (1.5)	WITH VEGETABLES
SALADS	10 (1.5)	9 (1.5)	BREADS WITH
*ULPO* WITH OR WITHOUT MILK	10 (1.5)	9 (1.5)	*PAVO DE HARINA TOSTADA*
RICE WITH...	9 (1.4)	9 (1.5)	FISH
SPANISH-STYLE TORTILLA	9 (1.4)	9 (1.5)	PASTAS
WATER WITH	8 (1.2)	8 (1.4)	BARBECUE
OATMEAL	8 (1.2)	8 (1.4)	MATÉ
LEFTOVERS FROM LUNCH	8 (1.2)	8 (1.4)	MARMALADE
COOKIES	8 (1.2)	8 (1.4)	SPANISH-STYLE TORTILLA
*MANJAR*, MARMALADE, HONEY	8 (1.2)	7 (1.2)	SALADS
CHICKEN	8 (1.2)	7 (1.2)	MILK-BASED DESSERTS
*SOPAIPILLAS ETC*	8 (1.2)	6 (1)	STOMACH/VICERAS
*POROTOS CON RIENDAS*	7 (1.1)	6 (1)	MILK
SANDWICH/HOT DOG	7 (1.1)	6 (1)	LEGUMES: FAVA BEANS
CEREALS	6 (0.9)	6 (1)	BUTTER OR LARD
MILK CHOCOLATE	6 (0.9)	6 (1)	*PASTEL DE CHOCLO/HUMITA*
*EMPANADAS*	6 (0.9)	6 (1)	SOPA DE PAN
MILK (COW’S)	6 (0.9)	6 (1)	*TORTILLA DE RESCOLDO*
OTHER ALGAES	6 (0.9)	5 (0.8)	*CHANGLES*
PIZZAS	6 (0.9)	5 (0.8)	LEFTOVERS FROM LUNCH
DISHES WITH *MOTE*	6 (0.9)	5 (0.8)	WITH *DIGUENES*
MILK-BASED DESSERTS	6 (0.9)	5 (0.8)	FLOUR WITH
WITH *COCHAYUYO*	5 (0.8)	5 (0.8)	JUICES
JUICES	5 (0.8)	5 (0.8)	BREADS: PROBABLY HOMEMADE
*PASTEL DE CHOCLO/HUMITA*	5 (0.8)	5 (0.8)	CREPES
VEGETABLES (1 TYPE) ^#^	5 (0.8)	5 (0.8)	*PANTRUCAS*
		5 (0.8)	*POROTOS CON RIENDAS*
		5 (0.8)	SALMON
		5 (0.8)	YOGURT

† Grey shading represents subcategories common to both regions. Here follow brief translations for traditional dishes: *Cazuelas*: liquid stock with different meat and vegetable ingredients added; *Ulpo*: beverage made of toasted flour; *Manjar*: caramel made of condensed milk; *Sopaipillas*: fried dough (originally made out of zucchini); *Porotos con riendas*: dish consisting of white beans, spaghetti noodles, and chorizo; *Empanadas:* baked or fried savory turnovers; *Mote*: wheat germ; *Cochayuyo:* kelp; *Pastel de choclo:* pie of mashed corn, with ground beef, chicken, or turkey and eggs; *Humitas*: mashed corn cakes; *Pavo de harina tostada*: similar to *Ulpo*, but with salt and animal fat added; *Sopa de pan*: soup broth with hard bread; *Tortilla de rescoldo*: bread cooked in ash; *Changles/diguenes:* mushrooms endemic to Chile; *Pantrucas*: dumpling soup with potatoes. ^#^ This subcategory includes vegetables such as “tomatoes” and “endemic mushrooms” that were not frequent enough to elicit their own subcategory such as in the AR.

**Table 5:** Short list of traditional culinary preparations ranked by sustainability score by region.

Metropolitan Region		Region of La Araucanía	
Culinary Preparation	Score	Culinary Preparation	Score
Fruit: whole or salad	91%	Salads	94%
Salads: *Chilean* or tomato or mixed	90%	Scrambled eggs	92%
Scrambled eggs w/tomato or onion	82%	Homemade bread/*tortilla de rescoldo*	90%
Tea, coffee or maté	81%	Tea, coffee	90%
Vegetable soup	78%	*Chilean* salad	87%
Legumes: lentils, beans or garbanzos	78%	Fruit: whole or salad	86%
Spanish style tortilla with vegetables	76%	Legumes: lentils	84%
*Porotos granados/con mazamorra* (beans w/corn)	75%	*Porotos con riendas* (Chilean dish w/white beans, spaghetti and sausage)	85%
Vegetable “pudding”/vegetable stew	72%	*Cazuela* (Chilean soup): chicken or beef	85%
*Cochayuyo* (algae) salad	72%	*Cochayuyo* (algae) salad or cochayuyo w/cooked potatoes	80%
Whole grain bread w/avocado or avocado w/chopped onion	72%	Legumes: garbanzos or peas	79%
*Charquican* (Chilean dish of ground beef, potatoes, corn)	70%	*Carbonada* (Chilean beef stew)	79%
Cheese w/tomato	69%	Maté	77%
*Causeo*: tomato w/garlic or tuna	69%	Chicken w/peas	76%
*Cazuela* (Chilean soup): chicken or beef	67%	Leftovers from lunch	75%
*Porotos con riendas* (Chilean dish w/white beans, spaghetti and sausage)	64%	Cooked fava beans	75%
Leftovers from lunch	61%	Cheese of any kind	74%
Juicy baked chicken	61%	*Pantrucas* (Chilean meat, noodle and potato stew)	74%
*Ulpo* (toasted wheat flour drink) w/or w/out milk	60%	Avocado	73%
*Pantrucas* (Chilean dumpling soup with potatoes)	58%	Boiled corn	73%
Ceviche or baked fish	55%	Homemade marmalade	72%
Fish soup	52%	*Humitas* (Chilean corn cakes)	71%
Shredded beef	48%	Homemade *Kugen* (any fruit)	68%
Empanadas	39%	*Pastel de choclo* (Chilean corn-based dish with ground beef, turkey, egg)	65%
		Soup with mussels or mixed seafood	63%
		Salad w/*diguenes* (Mushroom endemic to Chile)	61%
		Large pine nuts	54%

## Supplementary Materials

The following are available online at https://www.mdpi.com/2072-6643/12/2/489/s1. Figure S1: Traditional culinary preparations prepared during the key informant interviews.

## Appendix A

The brief protocol used to guide the community workshops. This provides the materials and methods sequence with recommendations for time management related to free listing and related activities in a community workshop setting in which only three hours is allowed per workshop.

**Community Workshops Protocol—Mini Guide**

**Key Preparation before a Community Workshop**

Review the free list data from the key informants (form 2.1) for background information that could be useful, such as the traditional culinary preparations listed by the age or ethnic group for the given community workshop coming up; and become familiar with the traditional culinary preparations that were most frequently mentioned during the free listing of the key informants, but those which lacked reference to mealtime. Use the community workshops to determine if any of these most frequently mentioned traditional culinary preparations by the key informants pertain to a specific mealtime or eating occasion. Materials: digital recorder with batteries, notebook, pen, informed consent forms (2.2–2.5 b), and envelopes with cash incentive.

**Before beginning the data collection process at a community workshop, remember to:** explain and obtain the written informed consent; verify personal data to assign unique identifiers; disseminate and have each participant sign for the receipt of the incentive; arrange the snacks and coffee; and put up the poster forms (2.2–2.3). *Remember to always fill out the data at the top and/or bottom of the forms to identify to which community workshop they belong.*

**I. Round 1: Forms 2.2–2.3 (the posters)—Planned time: 30 min**

Forms 2.2–2.3 First column (15 min): This part is very similar to the free listing of Form 2.1 from the key informant interviews. Suggested questions are:

- Do there still exist mealtimes such as breakfast, lunch, *once*, and/or dinner? What happens with regard to additional mealtimes, such as snack breaks (*colaciones*), especially amongst adults?
- Why was bread with avocado not mentioned? When do they eat empanadas? When do they eat fruit? When do they eat *charquicán* and other main dishes?
- Common themes from the key informant interviews: for example, does *sofrito/verdurita* (sautéed vegetable base for many recipes) also come up in the community workshops, or not so much?

Forms 2.2–2.3* the other columns (15 min): To categorize consumption frequency and seasonality, suggested questions are:

- Why is winter more frequently mentioned than summer, even though there are more fruits and vegetables in summer?

* If there is not enough space to record data on the two poster forms (2.2–2.3) these extra data should be recorded by hand on a blank (U.S. Letter size) Form 2.2 that the research assistant will have with them during the workshop.

**II. Round 2: Form 2.4—One (1) form for each preparation listed on the posters/forms 2.2–2.3—Planned time: 90 min**.

- An important point to clarify about the first part “Notes about the preparation”: Here does NOT ask for a complete recipe to be recorded. If you think there is a rare and/or special recipe for a given preparation amongst the group, *then you should fill out Form 2.5 b after the community workshop has ended.*
- *A reminder about Form 2.4 page 2:* Record the price tendencies for key ingredients of a preparation by month in the first table. For example, corn would have a low price in January, but corn would have high price in July. In the second table: record the relative importance of the use frequency or amount of consumption in general for the group by month.

**III. Round 3: Group discussion about taste and the Forms 2.5 (if applicable)—Planned time: 30 min**

- If the group is >65: Part 1—Form 2.5 ( < 15 min): Similar to Form 2.1 of the key informant interviews ****A rare preparation is a preparation that today, in the present, is practically not consumed, but memories still exist about this—now rare—preparation.
- If the group is not >65, BEGIN HERE: Part 2—Discussion about taste (10–30 min): A discussion about “taste”—between 0 or 7—of each preparation listed per row on the posters (forms 2.2–2.3).
- For example, if three people say that “they absolutely do not like that preparation,” then next to this row, write: 0–3 (*or just write in red marker the number of people, so a red 3).*
- For example, if 2 people say that “they absolutely love that preparation,” then next to this row, write: 7–2 (*or just write in green marker the number of people, so a green 2).*

**After the workshop: Form 2.5 b: rare recipes of preparations**
