# Supplementary material for: Participatory Methods to Identify Perceived Healthy and Sustainable Traditional Culinary Preparations across Three Generations of Adults: Results from Chile’s Metropolitan Region and Region of La Araucanía
Source: Nutrients. 2020 Feb 14;12(2):489. doi: 10.3390/nu12020489 (PMC7071181; doi:10.3390/nu12020489)
Supplement: Supplementary file 1 [file nutrients-12-00489-s001.pdf]

Supplementary Figure S1: Traditional culinary preparations prepared during the key informant interviews

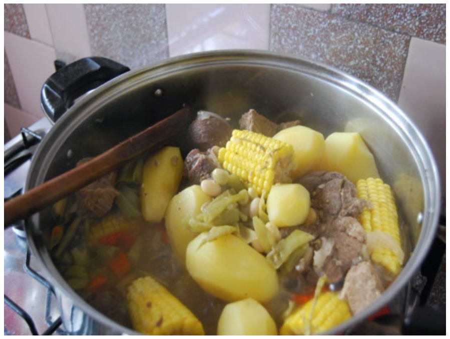

(**a**)

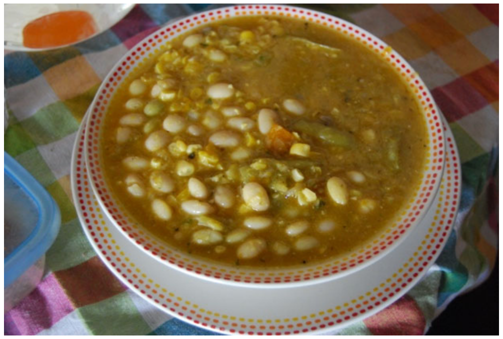

(**b**)

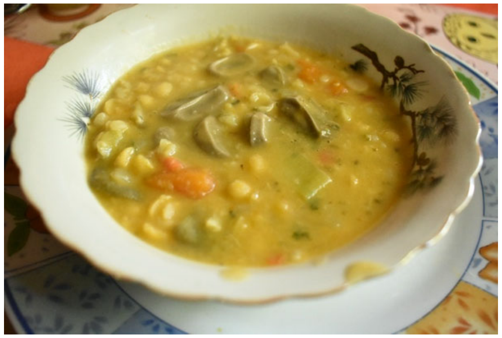

(f)

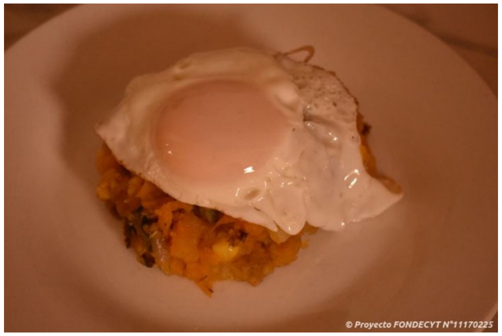

(g)

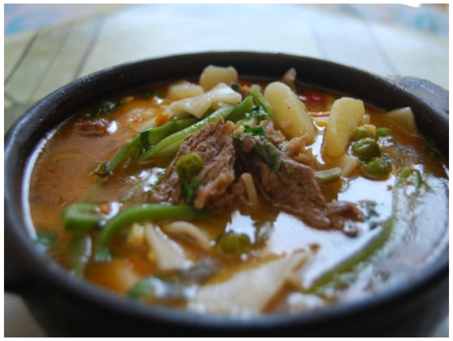

(c)

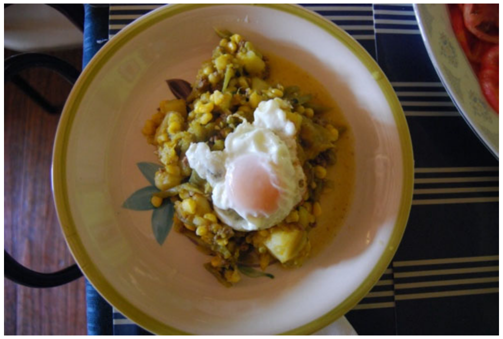

(d)

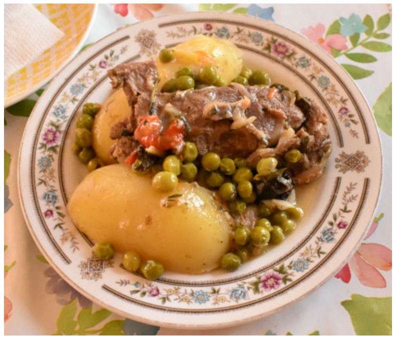

(h)

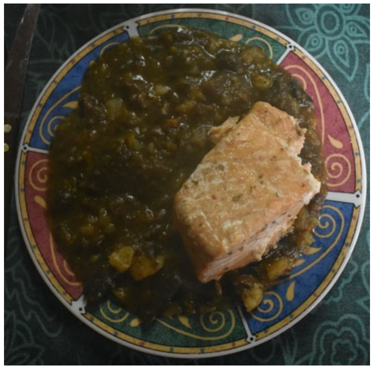

(i)

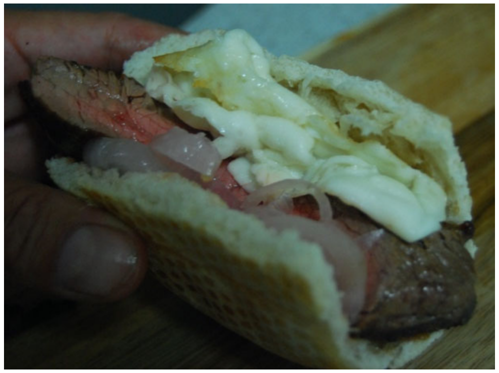

(**e**)

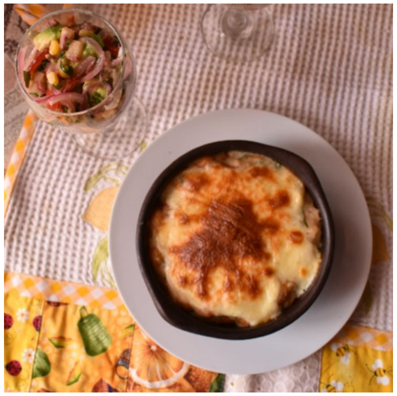

(**j**)

Supplementary Figure 1. The descriptions of the traditional culinary preparations are presented as follows: *Chilean Spanish name* [English translation] – strata. All photo credits to Mariana Léon (M.L.V.) and cannot be shared without permission. Panels a-e are from the Metropolitan Region: (**a**) *Hervido de trilla* [Boiled wheat] – Woman > 65 y, not First Nations; (**b**) *Porotos granados* [Bean stew] – Woman 45-65 y, not First Nations; (**c**) *Pantrucas* [Dumpling soup with potatoes] – Woman 25-45 y, not First Nations; (**d**) *Charquicán* [Stew dish with minced beef, potatoes, pumpkin, corn, onion and peas and topped with a fried egg] – Woman 25-45 y, First Nations; (**e**) *Sandwich de la cruz* [Sandwich with steak, cheese and onions] –Man 25-45 y, not First Nations; Panels f-j are from the Region of La Araucanía: (**f**) *Garbanzos con mote y habas* [Garbanzos with boiled wheat and fava beans] – Woman > 65 y, not First Nations; (**g**) *Charquicán* [Stew dish with charqui, potatoes, pumpkin, corn, onion and peas and topped with a fried egg] – Woman 45-65 y, not First Nations; (**h**) *Cordero Arvejado* [Lamb with peas] – Woman 45-65 y, not First Nations; (**i**) *Charquicán de cochayuyo* [Stew dish with algae, potatoes, pumpkin, corn, onion and peas; bread on the side] – Woman 45-65 y, First Nations; (**j**) *Chupe con Digueñes y Ensalada de Nalca* [Creamy stew made with *Digueñes* – an edible fungus native to South-Central Chile; and Chilean rhubarb salad] –Woman 25-45 y, First Nations.
